# Web-Based Fully Automated Self-Help With Different Levels of Therapist Support for Individuals With Eating Disorder Symptoms: A Randomized Controlled Trial

**DOI:** 10.2196/jmir.5709

**Published:** 2016-06-17

**Authors:** Jiska J Aardoom, Alexandra E Dingemans, Philip Spinhoven, Joost R van Ginkel, Mark de Rooij, Eric F van Furth

**Affiliations:** ^1^ Rivierduinen Eating Disorders Ursula Leiden Netherlands; ^2^ Institute of Psychology, Leiden University Leiden Netherlands; ^3^ Leiden University Medical Center Department of Psychiatry Leiden Netherlands; ^4^ Institute of Education and Child Studies, Leiden University Leiden Netherlands

**Keywords:** ehealth, eating disorders, Internet-based, therapist support, self-monitoring, self-help

## Abstract

**Background:**

Despite the disabling nature of eating disorders (EDs), many individuals with ED symptoms do not receive appropriate mental health care. Internet-based interventions have potential to reduce the unmet needs by providing easily accessible health care services.

**Objective:**

This study aimed to investigate the effectiveness of an Internet-based intervention for individuals with ED symptoms, called “Featback.” In addition, the added value of different intensities of therapist support was investigated.

**Methods:**

Participants (N=354) were aged 16 years or older with self-reported ED symptoms, including symptoms of anorexia nervosa, bulimia nervosa, and binge eating disorder. Participants were recruited via the website of Featback and the website of a Dutch pro-recovery–focused e-community for young women with ED problems. Participants were randomized to: (1) Featback, consisting of psychoeducation and a fully automated self-monitoring and feedback system, (2) Featback supplemented with low-intensity (weekly) digital therapist support, (3) Featback supplemented with high-intensity (3 times a week) digital therapist support, and (4) a waiting list control condition. Internet-administered self-report questionnaires were completed at baseline, post-intervention (ie, 8 weeks after baseline), and at 3- and 6-month follow-up. The primary outcome measure was ED psychopathology. Secondary outcome measures were symptoms of depression and anxiety, perseverative thinking, and ED-related quality of life. Statistical analyses were conducted according to an intent-to-treat approach using linear mixed models.

**Results:**

The 3 Featback conditions were superior to a waiting list in reducing bulimic psychopathology (d=−0.16, 95% confidence interval (CI)=−0.31 to −0.01), symptoms of depression and anxiety (d=−0.28, 95% CI=−0.45 to −0.11), and perseverative thinking (d=−0.28, 95% CI=−0.45 to −0.11). No added value of therapist support was found in terms of symptom reduction although participants who received therapist support were significantly more satisfied with the intervention than those who did not receive supplemental therapist support. No significant differences between the Featback conditions supplemented with low- and high-intensity therapist support were found regarding the effectiveness and satisfaction with the intervention.

**Conclusions:**

The fully automated Internet-based self-monitoring and feedback intervention Featback was effective in reducing ED and comorbid psychopathology. Supplemental therapist support enhanced satisfaction with the intervention but did not increase its effectiveness. Automated interventions such as Featback can provide widely disseminable and easily accessible care. Such interventions could be incorporated within a stepped-care approach in the treatment of EDs and help to bridge the gap between mental disorders and mental health care services.

**Trial Registration:**

Netherlands Trial Registry: NTR3646; http://www.trialregister.nl/trialreg/admin/ rctview.asp?TC=3646 (Archived by WebCite at http://www.webcitation.org/6fgHTGKHE)

## Introduction

Eating disorders (EDs) are serious psychiatric disorders characterized by high rates of comorbidity, chronicity, mortality, and relapse [1-5]. Unfortunately, despite the disabling nature of these disorders, many individuals with ED symptoms do not seek and receive appropriate mental health care [2,6]. Barriers to care include geographical or financial barriers, as well as fear of stigmatization and feelings of shame [7]. E-mental health has the potential to reduce these barriers in help-seeking, as well as the unmet need for health care by providing easily accessible services.

Numerous Internet-based interventions for the prevention and treatment of ED have shown promising results [8-10]. The results of a recent meta-analytic review [10] demonstrated that Internet-based programs, of which the majority was based on cognitive behavioral principles, were successful in decreasing a range of ED-related symptoms including body dissatisfaction, symptoms of bulimia nervosa, shape and weight concerns, dietary restriction, and negative affect. Emerging research furthermore suggests that ehealth interventions may reach underserved populations and increase access to regular health care [11]. Despite the promising results, research into the effectiveness of such interventions is still in an early stage [9,12,13], and further high-quality studies are required.

Internet-based interventions can include many different components and can be provided with or without therapist support. In the field of depression and anxiety, it has been found that Internet-based interventions with therapist support were more effective than those without or those with only minimal therapeutic contact [14,15]. Direct comparisons of Internet-based mental health interventions with and without therapist support in randomized controlled trials are scarce although a recent meta-analysis indeed demonstrated guided interventions to be superior to unguided interventions [16]. However, studies investigating the optimal intensity of therapist support are rare [16], and it is currently unknown how much or how little therapist support is needed to realize a particular amount of additional improvement in health outcomes. To our knowledge, only 1 study directly compared different intensities of therapist support in an Internet-based treatment for panic disorder [17]. This study demonstrated no significant differences between higher and lower intensities of therapist support. Regarding ehealth interventions in the field of ED, no studies have yet directly compared guided and nonguided interventions nor have different intensities of therapist support been investigated.

In addition to the intensity of therapist support, another important factor is the way in which such support is provided. Tate et al [18] investigated the effectiveness of feedback on self-monitoring diaries provided by either a human counselor or a computer-automated program in an Internet-based weight loss program. Interestingly, at 3-month follow-up, no significant differences in outcome were found between participants in the computer-automated counseling condition and the human counseling condition, respectively. Along similar lines, a recent study demonstrated a Web-based intervention for mild-to-moderate depression symptoms to be equally effective when provided with human versus automated support [19]. Hence, automated support may be an effective and widely disseminable means of providing support within Internet-based interventions, and it is important to further compare the effectiveness of such automated support with the effectiveness of different intensities of individual therapist support.

This study evaluated self-help intervention “Featback” for individuals with ED symptoms. Featback comprises psychoeducation and a fully automated self-monitoring and feedback system. Self-monitoring is an important clinical technique that is often used in cognitive behavioral therapy [20], where it can among other things help to gain a more comprehensive understanding of one’s psychopathology. By means of the monitoring and feedback system, participants are invited to complete a weekly monitoring questionnaire assessing the core symptoms of ED: body dissatisfaction, excessive concern with body weight and shape, unbalanced nutrition and dieting, and binge eating and compensatory behaviors. After completion of the questionnaire, participants receive a feedback message, which is automatically generated and tailored to their answers of the monitoring questions, containing social support and advice on how to counteract reported ED symptoms. Featback is aimed at individuals with all types of ED symptoms, which in line with the transdiagnostic theory that all EDs (eg, anorexia nervosa, bulimia nervosa, binge eating disorder) share the same core psychopathology, characterized by the overevaluation of eating, shape, weight, and their control [21].

The first aim of this study was to investigate the effectiveness of Featback in reducing ED psychopathology and comorbid symptoms. The second aim was to investigate the added value of therapist support and different intensities of therapist support. A randomized controlled trial was conducted comparing 4 conditions: (1) Internet-based intervention Featback, consisting of psychoeducation and a fully automated monitoring and feedback system, (2) Featback supplemented with low-intensity (weekly) therapist support, (3) Featback supplemented with high-intensity (3 times a week) therapist support, and (4) a waiting list control (WLC).

## Methods

### Study Design and Procedure

This study was a 4-arm randomized control trial. Ethical approval was obtained from the Leiden University Medical Center Ethics Committee. This committee granted exemption for parental consent for individuals aged between 16 and 18 years of age. Detailed information on the study methods, including the design, intervention conditions, measures, and ethical precautions and crisis management, can be found in the published study protocol [22].

Participants were recruited via the website of Featback [23] and the website of Dutch pro-recovery–focused e-community “Proud2Bme” [24] for young women with ED problems. The eligibility criteria were: (1) age ≥ 16 years, (2) access to the Internet, and (3) ED symptoms. The latter was defined as scoring ≥ 52 on the Weight Concern Scale [25] or reporting 1 or more of the following ED symptoms as assessed by the Short Evaluation of Eating Disorders (SEED) [26]: a body mass index of ≤18.5, ≥ 1 binge eating episodes a week over the past 4 weeks, and engagement in ≥ 1 compensatory behaviors a week over the past 4 weeks.

After Web-based completion of informed consent and the screening questionnaire including questions regarding the eligibility criteria, participants were invited to complete the baseline questionnaire. Thereafter, participants were randomly assigned to 1 of the 4 study conditions with a block size of 40 and an equal allocation ratio (1:1:1:1). An independent researcher who had no involvement in any other aspect of this study conducted the randomization allocation by means of computer-generated random numbers created in SPSS. She concealed the allocation sequence in a password-protected computer file from the main researchers until interventions were assigned, preventing researchers from having any prior knowledge of the upcoming condition assignments. Importantly, therapists were alternately assigned to low- versus high-intensity therapist support.

### Interventions

#### Featback

All participants had access to the Featback website where comprehensive and general information on ED could be found (ie, psychoeducation), for example, the types of EDs and symptoms, risk factors, causes, and comorbid problems. This information served primarily to educate participants about EDs and stimulate recognition and acknowledgement. The psychoeducation was purely self-guided, meaning that participants were free in choosing when and what to read. The monitoring and feedback system comprised a weekly invitation by email to complete a monitoring questionnaire. This questionnaire consisted of 8 4-point Likert items assessing cognitive and behavioral correlates of the following 4 dimensions: (1) body dissatisfaction, (2) excessive concerns with body weight and shape, (3) unbalanced nutrition and dieting, and (4) binge eating and compensatory behaviors. After completion, an algorithm determines the patterns of change of each of these 4 dimensions: still in the functional or healthy range, still in the dysfunctional or unhealthy range, improvement from the dysfunctional to the functional range, or deterioration from the functional to the dysfunctional range. The 4 different patterns of change with respect to the 4 dimensions of ED symptoms result in 4×4×4×4= 256 possible scenarios regarding a participant’s status. For each possible scenario, 10 to 15 different feedback messages were preformulated in a database. After determining the status of a participant, the algorithm randomly selected 1 tailored feedback message out of this database and sent this to the participant accordingly. Hence, when a participant’s status does not change over time, one would not receive the same message over and over again. All the feedback messages contained social support by expressing interest in and concerns about the participants’ well-being. Positive reinforcement techniques such as encouragement were used to stimulate and maintain healthy behaviors and attitudes. Furthermore, the messages included tips and advice on how to counteract negative developments in reported ED-related symptoms. The following is an example of a feedback message, which could be sent to someone with dysfunctional overconcerns with body weight and shape, unbalanced nutrition and dieting (dysfunctional), as well as deteriorations in body dissatisfaction and symptoms of binge eating and compensatory behaviors:

We are concerned with the changes in your body image and eating behaviors, however, we know that you have the ability to make healthy changes. Your body image and eating habits are closely linked. This week, try to eat regular, well-balanced meals and snacks, which might help to prevent the binge eating and/or compensatory behaviors and help you to feel better. If you continue to have negative thoughts about your body, it may be helpful for you to talk to someone about it, maybe a family member? Or a friend? Take care!

The fully automated self-monitoring and feedback system was developed in Germany, and for more detailed information on this system, see the study by Bauer et al [27]. A reminder was sent to participants by email each time they failed to complete a monitoring assessment.

#### Featback + Low-Intensity Therapist Support

Participants received Featback as described previously supplemented with low-intensity (weekly) therapist support by means of email, chat and/or audio teleconference (ie, Skype). Participants could schedule support sessions in a Web-based agenda where available time slots of the therapist were presented. For each support session, participants could choose their preferred medium of support. Therapists were instructed to send an email to participants in case they did not schedule any support session(s) or in case they did not show up at scheduled support session(s) and to repeat this process twice per nonresponse. Chat and teleconference sessions had a maximum duration of 20 minutes, whereas an email session contained 1 email reply from the therapist to the participant. The therapist support was independent of the monitoring and feedback system. The chat methodology was based on a 5-phase model: (1) a warm welcome, (2) clarifying the question, (3) determining the goal of the conversation, (4) concrete elaboration of the goal of the conversation, and (5) closing the circle [28]. The email methodology contained 3 phases: (1) extracting the question, (2) formulating an answer, and (3) checking and rereading the message and sending it [28].

#### Featback + High-Intensity Therapist Support

Participants received Featback, supplemented with high-intensity (3 times a week) therapist support by means of email, chat, and/or teleconference as described previously.

#### Waiting List Control Condition

Participants were placed on a waiting list for 5 months, after which they were offered Featback with low-intensity therapist support.

In all 4 intervention conditions, participants were free to undergo any other type of intervention or treatment (ie, usual care).

#### Therapists

The therapists were 7 females who were either Master of Science students in clinical psychology or individuals with a master’s degree in clinical psychology. All therapists underwent training in the delivery and methodology of Internet-based support. Furthermore, they received extensive information on EDs and practiced with case material and expert patients (ie, someone who has experienced an ED themselves and has been successful in managing the disorder) before the start of the trial. Monthly face-to-face supervision sessions were organized by the main researcher (JA), a psychologist (MN), and an experienced psychotherapist (EvF) as a matter of routine professional and ethical care, as well as to reinforce adherence to the protocol. In addition, 2 individual supervision sessions were provided to all therapists during their first month. Thereafter, therapists’ adherence to the protocol was regularly checked at random, by checking whether the chats and emails included the 5- and 3-phase model, respectively.

### Outcomes

All data were collected by means of Internet-administered self-report questionnaires at baseline, post-intervention (8 weeks after baseline), and at 3- and 6-month follow-up. Waiting list participants were offered Featback with low-intensity therapist support after the 3-month follow-up and were not assessed at 6-month follow-up.

The primary outcome measure was ED psychopathology as measured by the SEED [26] and the Eating Disorder Examination Questionnaire (EDE-Q) [29]. The SEED [26] distinguishes between the main symptoms of anorexia nervosa (underweight, fear of weight gain, distortion of body perception) and bulimia nervosa (binge eating, compensatory behaviors, overconcern with body shape and weight). Total severity indexes were calculated for both dimensions. The SEED has demonstrated validity and was shown to be sensitive to symptom change [26]. Regarding the EDE-Q, a global score of ED psychopathology was calculated by summing and averaging 22 7-point Likert items. The EDE-Q has demonstrated reliability and validity [30], and the internal consistency reliability in the current sample was high (Cronbach α=.88). Higher scores on both the SEED (range 0-3) and the EDE-Q (range 0-6) reflect higher ED psychopathology.

Secondary outcome measures included ED-related quality of life as assessed by the ED-Related Quality of Life Questionnaire (ED-QOL), a validated 25-item questionnaire assessing the influence of eating behaviors and body weight in the psychological, physical and cognitive, financial and work- or school-related domain [31]. The ED-QOL demonstrated excellent internal consistency reliability in this study sample (Cronbach α=.92). Higher scores (range 1-5) reflect lower quality of life. Symptoms of depression and anxiety were measured using the 4-item Patient Health Questionnaire (PHQ-4). The PHQ-4 has demonstrated factorial and construct validity [32] and demonstrated good internal consistency reliability in the current sample (Cronbach α=.83). Higher scores (range 0-12) reflect higher symptom severity. Finally, levels of perseverative thinking (ie, worry and rumination) were assessed using the Perseverative Thinking Questionnaire (PTQ) [33]. The PTQ demonstrated good internal consistency and satisfactory stability [33]. The internal consistency reliability in the current sample was excellent (Cronbach α=.95). Higher scores are indicative of higher levels of perseverative thinking (scale 0-4).

Given that participants were free to undergo any other type of intervention, psychological health care service utilization (ie, appointments with a dietitian, social worker, psychologist, psychiatrist, or psychotherapist) was assessed with the Trimbos/iMTA Questionnaire for Costs Associated with Psychiatric Illness: TiC-P) [34]. User satisfaction was assessed with 2 open-ended questions asking participants for their positive and negative feedback, respectively. In addition, participants were asked to rate their satisfaction with the intervention and their satisfaction with their therapist on a 10-point Likert scale ranging from very dissatisfied (score of 1) to very satisfied (score of 10). Finally, 2 open-ended questions assessed the reasons for dropout attrition (ie, not completing study questionnaires) and nonusage attrition (ie, deregistration from the monitoring and feedback system).

### Statistical Analyses

All data were analyzed in SPSS version 22 using 2-tailed tests and α=.05. A target sample size of 344 participants was calculated by the software program Power Analysis and Sample Size version 8.0 (2008) to yield 80% power to detect an expected between-group (pooled Featback conditions vs WLC) difference at post-intervention with an effect size of 0.3, α=.05, and an expected dropout rate of 30% (for more details on power calculation, see the paper by Aardoom et al [22]).

Possible differences in baseline characteristics, dropout rates, and participants’ experiences were investigated using chi-square tests and analysis of variances. All data were imputed using multiple imputation methods. Multiple imputations using predictive mean matching were conducted in statistical software program R version 3.02. Interactions were taken into account in the imputation procedure [35]. Multiple imputation methods have several advantages over complete-case analyses or single imputation techniques and are therefore highly recommended [36]. For each variable with missing data, the number of predictor variables was determined by the rule of thumb of 15 cases per potential predictor [37]. For example, in case the data of 300 participants would be available on a specific variable, 300/15=20 predictor variables could be used to predict missing data on this variable. Then, correlations between the outcome variable and all other variables were investigated, so that the variables that correlated the highest with the outcome variable were chosen as predictors for the missing data on the outcome variables. A total of 100 imputed datasets were generated. Results from all imputed datasets were pooled according to Rubin’s rules to account for the uncertainty associated with the imputations [38].

The main analyses were conducted using linear mixed models including random intercepts. All analyses were conducted according to the intent-to-treat approach including all participants who underwent randomization. Three statistical models were specified including time and condition contrasts (for details on models and contrast coding, see Multimedia Appendix 1). Model 1 investigated whether the 3 Featback conditions (pooled) led to better outcomes than the WLC. Model 2 compared Featback without therapist support versus the 2 Featback conditions with therapist support (pooled). Model 3 compared Featback with low- versus high-intensity therapist support. Main analyses were repeated controlling for significant baseline differences between the conditions (ie, age, marital status, and duration of ED psychopathology), and number of received psychological health care appointments. The latter was entered as covariate to examine intervention effects over and above usual care. Also, main analyses were repeated for completers of the intervention only, defined as participants who completed at least 5 monitoring questionnaires (Featback without therapist support), plus at least 5 to 13 therapist support sessions (Featback with low- vs high-intensity therapist support, respectively).

Effect sizes (d) were calculated by dividing the unstandardized coefficients of interaction effects (time × condition) by the pooled within-group standard deviation (SD) of the outcome measure at baseline [39]. The resulting effect sizes of all imputed dataset were summed and averaged. The 2 open-ended questions related to satisfaction with the intervention, both critical and positive, were qualitatively explored to provide an overview of participants’ most frequently reported negative and positive comments.

## Results

### Participants

**Figure 1:**
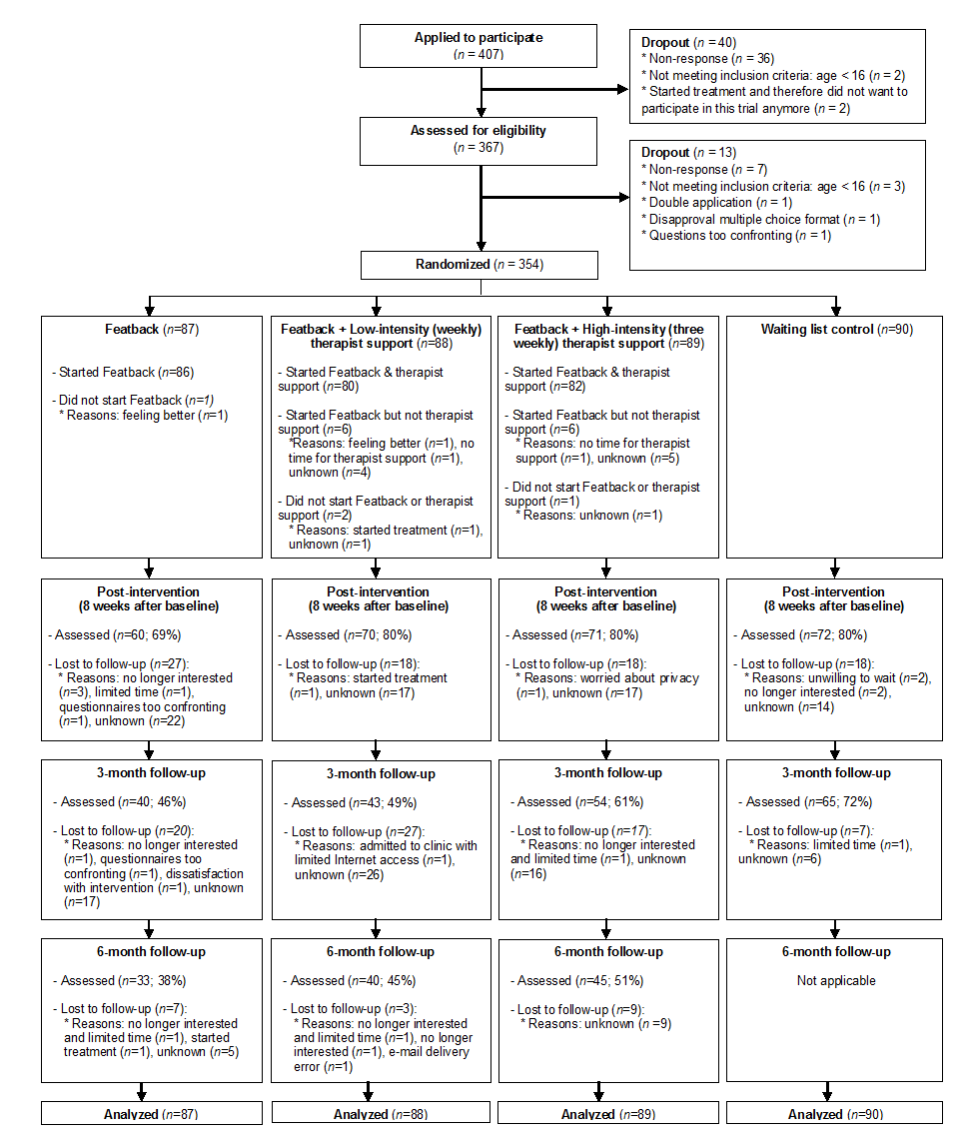
CONSORT diagram: Flow of participants through each stage of the randomized controlled trial.

Participants were recruited between November 7, 2012 and June 17, 2013. Follow-up was completed at March 3, 2014. Figure 1 presents the flow of participants through each stage of the trial. A total of 354 participants were assessed at baseline, 273 (77.1%) at post-intervention, 202 (57.1%) at 3-month follow-up, and 118 participants (44.7%) of the available 3 study conditions (n=264) at 6-month follow-up. Study dropout rates did not significantly differ between the conditions at post-intervention (*χ*^2^(3)=4.35, *P*=.23) and 6-month follow-up (*χ*^2^(2)=2.87, *P*=.24), although at 3-month follow-up, the WLC participants dropped out of the study less often than participants who received Featback without or with low-intensity therapist support (*χ*^2^(3)=15.69, *P*=.001). No differences in non-usage attrition were found among the 3 Featback conditions (*χ*^2^(2)=5.24, *P*=.07).

Baseline characteristics of participants are summarized in Table 1. Significant differences between the conditions were found regarding age, duration of ED psychopathology, and marital status, whereas no significant differences were found for any other baseline variables. No significant differences between the study conditions were found regarding the number of psychological health care appointments received (ie, appointments with a dietitian, social worker, psychologist, psychiatrist, or psychotherapist) during the intervention period (F(3,245)=0.29, *P*=.84). One hundred participants (40.2%) did not receive any psychological health care appointments during this period, whereas 149 participants (59.8%) did have such appointments (range 1-40).

**Table 1:** Baseline characteristics (nonimputed) of the study population; data are provided as means (SD) or numbers (percentages)

Characteristics		Featback (n=87)	Featback+ low-intensity therapist support (n=88)	Featback+ high-intensity therapist support (n=89)	Waiting list control condition (n=90)	Total sample (n=354)	Statistics
**Gender**							*χ* ^2^(3)=2.02, *P*=.57
	Male	1 (1.1%)	1 (1.1%)	2 (2.2%)	0 (0.0%)	4 (1.1%)	
	Female	86 (98.9%)	87 (98.9%)	87 (97.8%)	90 (100.0%)	350 (98.9%)	
**Marital status**							*χ^2^* (6)=13.22, *P*=.04
	Married or living together	28 (32.2%)^a,b^	17 (19.3%)^a,b^	21 (23.6%)^b^	11 (12.2%)^a^	77 (21.8%)	
	Single or living alone	58 (66.7%)	71 (80.7%)	67 (75.3%)	79 (78.8%)	275 (77.7%)	
	Divorced	1 (1.1%)	0 (0.0%)	1 (1.1%)	0 (0.0%)	2 (0.6%)	
**Education level**							*χ^2^* (6)=7.69, *P*=.26
	Low	4 (4.6%)	4 (4.5%)	7 (7.9%)	10 (11.1%)	25 (7.1%)	
	Intermediate	16 (18.4%)	26 (29.5%)	19 (21.3%)	17 (18.9%)	78 (22.0%)	
	High	67 (77.0%)	58 (65.9%)	63 (70.8%)	63 (70.0%)	251 (70.9%)	
**Use of psychotropic medication**							*χ^2^* (3)=3.35, *P*=.34
	Yes	21 (24.7%)	17 (19.5%)	16 (18.2%)	25 (28.4%)	79 (22.7%)	
	No	64 (75.3%)	70 (80.5%)	72 (81.8%)	63 (71.6%)	269 (77.3%)	
**Employment status**							*χ^2^* (9)=8.96, *P*=.44
	School or study	50 (58.1%)	48 (55.2%)	40 (45.5%)	51 (56.7%)	189 (53.8%)	
	Employed	25 (29.1%)	22 (25.3%)	35 (39.8%)	30 (33.3%)	112 (31.9%)	
	Unemployed or homemaker	4 (4.7%)	8 (9.2%)	4 (4.5%)	3 (3.3%)	19 (5.4%)	
	Sick leave or disabled	7 (8.1%)	9 (10.3%)	9 (10.2%)	6 (6.7%)	31 (8.8%)	
**Treatment history for ED^c^**							*χ^2^* (3)=4.43, *P*=.22
	Yes	48 (55.2%)	40 (45.5%)	39 (43.8%)	36 (40.0%)	163 (46.0%)	
	No	39 (44.8%)	48 (54.5%)	50 (56.2%)	54 (60.0%)	191 (54.0%)	
**Age (years)**		24.7 (7.1)^a,b^	23.0 (7.0)^a^	26.3 (9.2)^b^	22.8 (6.6)^a^	24.2 (7.7)	F(3,350)=4.17, *P*=.01
**Body mass index**		21.8 (5.0)	21.2 (4.8)	21.4 (5.4)	20.6 (4.6)	21.2 (5.0)	F(3,347)=1.03, *P*=.38
**Duration of ED problems (years)**		8.1 (6.9)^a,b^	6.5 (5.8)^a,b^	8.2 (7.7)^b^	5.7 (5.6)^a^	7.1 (6.6)	F(3,346)=3.05, *P*=.03
**Global ED psychopathology (EDE-Q)^d^**		4.2 (0.8)	4.4 (0.9)	4.0 (0.8)	4.1 (1.1)	4.2 (0.9)	F(3,113)=1.54, *P*=.21
**AN^e^psychopathology (SEED^f^-AN)**		1.1 (0.4)	1.1 (0.4)	1.1 (0.4)	1.1 (0.4)	1.1 (0.4)	F(3,347)=0.24, *P*=.87
**BN^g^psychopathology (SEED-BN)**		1.4 (0.7)	1.5 (0.7)	1.5 (0.6)	1.5 (0.7)	1.5 (0.7)	F(3,349)=0.30, *P*=.82

^a,b^Significant group differences were further investigated using Bonferroni post-hoc comparisons; different superscript letters indicate significant differences between the conditions.

^c^ED: eating disorder.

^d^EDE-Q: Eating Disorder Examination Questionnaire.

^e^AN: anorexia nervosa.

^f^SEED: Short Evaluation of Eating Disorders.

^g^BN: bulimia nervosa.

Participants in this study demonstrated severe levels of ED psychopathology: their EDE-Q scores were comparable to the overall norm for treatment-seeking patients with an ED in our clinical program [40]. The mean EDE-Q score of 4.2 (SD=0.9) is furthermore markedly above the clinical threshold, as recent literature demonstrated reliable EDE-Q cutoff scores of >2.50 [41] and >2.12 [42]. Approximately 98.6% (n=349) of the study participants scored above the cutoff score of 2.5. To provide a diagnostic impression of the study sample, we used the EDE-Q to approximate diagnostic classifications according to the fifth edition of the Diagnostic and Statistical Manual of Mental Disorders (DSM-5) [43]. Subsequently, 103 (29%) participants demonstrated symptoms of anorexia nervosa, being a body mass index of ≤18.5 combined with a fear of weight gain or of becoming fat. A total of 93 participants (26%) reported binge eating disorder symptoms: binge eating episodes once a week or more during the past 28 days, without recurrent use of inappropriate compensatory behaviors (ie, less than once a week over the past 28 days). Seventy-seven (22%) participants reported symptoms of bulimia nervosa, being episodes of binge eating and inappropriate compensatory behaviors both at least once a week or more during the past 28 days. Only 14 participants (4%) demonstrated symptoms of purging disorder, that is, purging behaviors once a week or more during the past 28 days in the absence of binge eating episodes. Finally, 5 participants (14%) reported ED symptoms that may be classified as “unspecified feeding or ED,” or ED problems without a DSM-5 classification. Seventeen participants (5%) could not be classified owing to missing data regarding binge eating episodes or body mass index. The 4 study conditions did not differ with respect to the type of ED (*χ*^2^(15)=19.33, *P*=.20).

#### Intervention Compliance

Participants in the 3 Featback conditions completed a mean number of 5.6 (SD=2.3, range 0-8) of 8 weekly monitoring questionnaires, with no significant difference between the conditions (F(2,261)=1.36, *P*=.258). Participants in the 2 Featback conditions with therapist support received a total of 1407 support sessions, with email being the most popular medium (n=937, 67%), followed by chat (n=417, 30%) and teleconference (n=53, 4%). These proportions of email (*t* (1,155)=−1.63, *P*=.11), chat (*t* (1,153)=1.42, *P*=.16), and teleconference (*t* (1,159)=0.53, *P*=.59) were similar for the 2 study conditions. The mean number of received therapist support sessions differed significantly between Featback with low- and high-intensity therapist support (*t* (175)=8.24, *P*<.001): participants in the former condition received on average 4.7 (SD=2.7, range 0-8) sessions, whereas participants in the latter condition received on average 11.2 (SD=6.9, range 0-24) sessions. Thus, we successfully created 2 different intervention conditions regarding the intensity of therapist support.

#### Comparison of Intervention Conditions With Waiting List Condition

The outcome data for each of the 4 conditions over time can be found in Multimedia Appendix 2. Table 2 summarizes the results of the mixed model analyses comparing the 3 Featback conditions with the WLC (statistical model 1). As summarized in Table 2, from baseline to post-intervention, significant time-by-condition effects were found for bulimic psychopathology (d=−0.16, 95% CI=−0.31 to −0.01), symptoms of depression and anxiety (d=−0.31, 95% CI=−0.54 to −0.09), and perseverative thinking (d=−0.28, 95% CI=−0.45 to −0.11). These interaction effects indicated greater reductions in psychopathology for participants in the Featback conditions as compared with the WLC. For global ED psychopathology and ED-related quality of life, only significant time effects were found, indicating improvements over time. From post-intervention to 3-month follow-up, significant time-by-condition effects were found for ED-related quality of life (d=−0.22, 95% CI=−0.38 to −0.06) and symptoms of depression and anxiety (d=−0.21, 95% CI=−0.33 to −0.09), indicating more improvements in the Featback conditions as compared with the WLC during the 3-month follow-up period (see Table 2). For anorectic and bulimic psychopathology and levels of perseverative thinking, no interaction effects were found, but significant time effects were found that indicated improvements over time. Completer analyses confirmed the conclusions of the intent-to-treat analyses and are therefore not reported.

#### Comparison of Active Intervention Conditions

In statistical models 2 and 3, we compared the intervention conditions and thus investigated the added value of therapist support, and higher versus lower intensities of therapist support, respectively. As shown in Multimedia Appendices 3 and 4, participants in all Featback conditions

**Table 2:** Results of linear mixed model analyses comparing the effectiveness of an Internet-based fully automated monitoring and feedback intervention with a waiting list control condition; results are based on the pooled results of 100 multiple imputed datasets.

Measure		Time effects			Time × condition effects		
		*B*	*t (P)*	95% CI	*B*	*t (P)*	95% CI
**Anorectic psychopathology (SEED^a^-AN^b^)**							
	Baseline to post-intervention	−0.02	−0.42 (.44)	−0.06 to 0.03	0.01	0.35 (.73)	−0.04 to 0.06
	Post-intervention to 3-month follow-up	−0.05	−2.21 (.03)	−0.10 to −0.006	0.04	1.41 (.16)	−0.02 to 0.09
**Bulimic psychopathology (SEED-BN^c^)**							
	Baseline to post-intervention	−0.07	−1.50 (.11)	−0.15 to 0.02	−0.11	−2.13 (.03)	−0.21 to −0.009
	Post-intervention to 3-month follow-up	−0.12	−2.51 (.01)	−0.22 to −0.03	−0.02	−0.42 (.67)	−0.14 to 0.09
**Global ED psychopathology (EDE-Q^d^)**							
	Baseline to post-intervention	−0.22	−3.07 (.002)	−0.37 to −0.08	−0.09	−1.08 (.28)	−0.26 to 0.08
	Post-intervention to 3-month follow-up	−0.18	−2.44 (.02)	−0.32 to −0.03	−0.07	−0.77 (.44)	−0.25 to 0.11
**ED-related quality of life (ED-QOL^e^)**							
	Baseline to post-intervention	−0.13	−3.46 (.001)	−0.20 to −0.06	−0.03	−0.74 (.46)	−0.12 to 0.05
	Post-intervention to 3-month follow-up	−0.06	−1.44 (.15)	−0.14 to 0.02	−0.13	−2.70 (.007)	−0.23 to −0.04
**Symptoms anxiety & depression (PHQ-4^f^)**							
	Baseline to post-intervention	−0.37	−1.92 (.06)	−0.74 to 0.007	−0.94	−4.11 (<.001)	−1.39 to −0.49
	Post-intervention to 3-month follow-up	−0.29	−1.43 (.15)	−0.69 to 0.11	−0.62	−2.53 (.01)	−1.11 to −0.14
**Perseverative thinking (PTQ^g^)**							
	Baseline to post-intervention	−0.08	−1.48 (.14)	−0.18 to 0.03	−0.20	−3.20 (.001)	−0.32 to −0.07	
	Post-intervention to 3-month follow-up	−0.16	−2.89 (.004)	−0.26 to −0.05	−0.05	−0.82 (.41)	−0.18 to 0.07

^a^SEED: Short Examination of Eating Disorders.

^b^AN: anorexia nervosa.

^c^BN: bulimia nervosa.

^d^EDE-Q: Eating Disorder Examination Questionnaire.

^e^ED-QOL: Eating Disorder–related Quality Of Life.

^f^PHQ-4: 4-item Patient Health Questionnaire.

^g^PTQ: Perseverative Thinking Questionnaire.

improved over time (baseline vs post-intervention, and post-intervention vs 3- and 6-month follow-up, respectively) with respect to bulimic psychopathology, global ED psychopathology, ED-related quality of life, symptoms of depression and anxiety, and levels of perseverative thinking (all *P* values ≤.01). When comparing Featback without therapist support with the pooled Featback conditions with therapist support (statistical model 2), the results demonstrated no significant differences between the conditions over time (all *P* values >.05, see Multimedia Appendix 3), indicating that participants improved to a similar degree. When comparing Featback with low- versus high-intensity therapist support (statistical model 3), no significant time-by-condition effects were found for most of the outcome measures (Multimedia Appendix 4). Except for ED-related quality of life, participants who received Featback with high-intensity therapist support showed greater improvements in ED-related quality of life from baseline to post-intervention (*P*=.001, d=0.15, 95% CI=0.06-0.24) and from post-intervention to 6-month follow-up (*P*=.01, d=0.14, 95% CI 0.03-0.25) than participants who received Featback with low-intensity therapist support. This finding should be interpreted with caution because participants who received Featback without therapist support scored in between and thereby not significantly different from the 2 Featback conditions with therapist support (Multimedia Appendix 2). Completer analyses confirmed the conclusions of the intent-to-treat analyses and are therefore not reported.

### Participants’ Experiences

Regarding participants’ experiences, significant differences in participants’ level of satisfaction with Featback were found (F(2,184)=38.41, *P*<.001). Participants who received Featback without therapist support were significantly less satisfied (M=5.0, SD=1.9, scale 1-10) than participants who received Featback with low- (M=7.1, SD=1.5) or high-intensity therapist support (M=7.4, SD=1.3), whereas no differences between the latter 2 were found. Overall, participants were very satisfied with the therapist support (M=8.0, SD=1.4, scale 1-10), with no significant differences between the low- and high-intensity therapist support conditions (*t* (1,117)= −0.34, *P*=.74). In addition, no significant differences in satisfaction with the different therapists were found (F(6,112)=0.36, *P*=.902).

A total of 158 participants provided negative feedback, and 160 participants provided positive feedback to the open-ended questions regarding their satisfaction with the intervention. Participants’ most reported critical comments included statements about the limitations of the automated feedback (n=95, 60%), for example, it being too general or impersonal, as well as the lack of more personal or individual therapist support. Most of the positive comments (n=107, 84.3%) included complementary remarks regarding the individual therapist support, such as participants having received good advice and support, having enjoyed the empathy, warmth, and attention of the therapists, as well as the feeling that someone was looking after them. Approximately one third (n=45, 28%) of all positive comments included positive feedback on this system, for example, experiencing the system as a good checkup supporting moments of reflection. No adverse effects from Featback were reported.

## Discussion

To our knowledge, this is the first randomized controlled trial to investigate an Internet-based fully automated self-monitoring and feedback intervention (Featback) and the added value of 2 different intensities of therapist support for individuals with ED psychopathology. The results demonstrated Featback to be superior to a WLC in reducing bulimic psychopathology (ie, a total severity index of binge eating, compensatory behaviors, and overconcern with body shape and weight), perseverative thinking, and symptoms of depression and anxiety. Thus, self-monitoring of ED-related attitudes and behaviors and receiving feedback by means of an automatic system can be effective in reducing psychopathology. No effects were found regarding anorectic psychopathology; hence, Featback may be more suitable for individuals with bulimic psychopathology. Interestingly, when comparing Featback with and without therapist support, no added value was found for therapist support in terms of the effectiveness of the intervention, although participants who received Featback with therapist support were significantly more satisfied.

Our findings add to the growing body of literature indicating the potential of ehealth interventions for individuals with (ED) psychopathology [8,9,12,13,44]. Our results are furthermore in line with 2 studies demonstrating that interventions supplemented with automated support can be equally effective to human support [18,19]. A fully automated Internet-based intervention such as Featback is a promising, widely disseminable, easily accessible, and potentially effective means of providing care for individuals with ED psychopathology. Such care is particularly important for these individuals, given that many do not seek or receive appropriate mental health care [6]. Hence, Internet-based self-help interventions might help to bridge the gap between mental disorders and mental health care services, by improving the help-seeking pathways. Internet-based automated self-monitoring and feedback systems may be of interest to a number of *other* areas in the field of psychiatry. Indeed, a recent study [45] demonstrated an Internet-based intervention including self-monitoring via text messages to be effective in remitted patients with symptoms of depression.

The finding that Featback was equally effective with and without therapist support is in line with that of several previous studies [46-48], however, in contrast to the result of a recent meta-analysis that included Internet-based interventions for a range of mental health problems [16]. This meta-analysis demonstrated guided Internet-based interventions to be significantly superior to unguided interventions. However, the larger effect sizes in the guided interventions may have been biased by significantly higher adherence rates in the guided interventions as compared with unguided interventions [16], whereas adherence rates in our study were similar for the guided and unguided conditions. A possible explanation for why therapist support did not enhance the effectiveness of Featback is that the monitoring and feedback system alone was already a relatively powerful intervention in reducing ED symptoms. Self-monitoring is an important clinical technique that is often used in cognitive behavioral therapy [20]. It can help an individual to gain a more comprehensive understanding of one’s psychopathology. By self-monitoring one’s psychopathology and receiving feedback, an individual is stimulated to think about the frequency, antecedents, and consequences of their problematic behaviors and attitudes [20]. Furthermore, through the provided feedback, individuals are encouraged to think about possible solutions to achieve positive behavioral changes, and in addition, the feedback can help them in applying and developing certain skills to promote such behavioral changes in their daily lives. It could be speculated that the self-monitoring and feedback system of the Featback intervention already provided such a powerful intervention to help reduce ED psychopathology that the therapist support did not add an extra effect. Within this context, the individual therapist support might primarily be appreciated for its empathy, warmth, and attention, as well as the feeling that someone is looking after you and listening to you.

Increasing the frequency of therapist support did not significantly affect outcome, which is in line with the results of a study that experimentally investigated different intensities of therapist support in an Internet-based treatment for panic disorder [17]. More frequent therapist support did furthermore not affect the participants’ satisfaction with the intervention or their therapist. Thus, increasing the amount of therapist contact may not necessarily result in increased effectiveness or increased satisfaction with Internet-based interventions. Nevertheless, future dose-response studies should replicate these rather unexpected findings before any firm conclusions can be drawn with respect to the added value of different intensities of therapist support. Also, cost-effectiveness studies comparing different intensities of therapist support would be of great interest. Such studies can facilitate decision making on how to most optimally deliver therapist support within Internet-based interventions. How much money needs to be invested in terms of additional therapist support to realize a particular amount of additional improvement in health outcomes? And does the extra benefit resulting from therapist support justify the extra cost: is adding a certain amount of therapist support good value for money?

Interestingly, our results show a discrepancy between the added value of therapist support in terms of effectiveness (no added value of therapist support) and satisfaction with the intervention (added value of therapist support). The fact that therapist support did increase the satisfaction of participants significantly might well be due to the empathy, warmth, and attention of the therapists. Individuals with ED are often ashamed about their ED and can feel isolated and unsupported, as well as misunderstood by their personal environment [49]. Although the automated feedback as part of Featback expresses interest in participants’ well-being and provides advice on how to possibly counteract certain dysfunctional beliefs or behaviors, it is not interactive. That is, individuals are not able to share their personal story, history, in-depth feelings and emotions, or experiences. In the individual therapist support sessions, they were able to (anonymously) ventilate their problems and emotions, and the majority reported on how nice it was to have someone looking after them, understanding them, and listening to them. Translating these study results to everyday clinical practice is challenging, given the added value of therapist regarding satisfaction, but not effectiveness. The resulting dilemma is about how to implement Featback: with or without therapist support? Adding such support implies more costs while not necessarily resulting in increased effectiveness. That being said, adding therapist support presumably heightens the attractiveness and thus reach of the intervention, eventually leaving more individuals feeling supported. An interesting future research direction would be to investigate the effectiveness of adding personal support by means of a Web-based peer support group. Possibly, the personal interactive support of peers might be sufficient to increase satisfaction rates, while at the same time reducing costs in comparison to trained professionals.

Adding therapist support did not enhance study adherence because no differences between study dropout rates were found between the 3 Featback conditions. However, our results showed that at 3-month follow-up, participants in the waiting list condition dropped out less often than participants who received Featback without or with low-intensity therapist support. Presumably, participants in the waiting list condition were more motivated to complete the study questionnaires given their knowledge that they would receive Featback with low-intensity therapist support after completing this follow-up questionnaire.

It is noteworthy that Featback produced significant reductions in psychopathology over and above usual care. Participants’ treatment status (yes or no) or number of received psychological health care appointments during the intervention period did not significantly differ between the study conditions and could furthermore not account for the superiority of Featback in comparison to WLC when entered as a predictor in the model. This suggests minimal self-help interventions such as Featback to be of interest for a broad population of individuals with ED symptoms. The small effect sizes match our expectations, given the type of intervention (ie, self-help) and the fact that most participants received psychological health care during the intervention period. Interventions such as Featback could be incorporated within regular treatment settings (ie, blended care), where it would enable accurate monitoring of patients’ well-being in treatment settings and in their everyday lives [20,50]. Also, information about patterns of dysfunctional attitudes and behaviors as gathered by the use of self-monitoring may aid in clarifying the rationale and goals for treatment, as well as informing therapists and patients about the patient’s progress in treatment. It could furthermore be useful to incorporate self-help interventions such as Featback as a first step within a stepped-care approach in the treatment of ED, thereby providing low-intensity care to individuals with ED symptoms who might not (yet) need more intense specialist care. Individuals who remain symptomatic after a certain period of time could then “step up” to a more intense specialist care. Similarly, Featback could also be used as a “step down” intervention after a more intensive treatment. Individuals can keep track of their ED symptoms and can be supported in their process of recovery. In addition, Featback as a “step down” intervention could allow for early identification and prevention of relapse. The potential effectiveness and cost-effectiveness of a stepped-care approach starting with self-help, as compared with cognitive behavior therapy, has already been demonstrated in a large multicenter trial for individuals with bulimia nervosa [51,52]. In sum, investigating the effectiveness of Featback within treatment settings, or as part of stepped-care approaches in the treatment of ED, is an interesting area for future research.

This study has several strengths and limitations. Strengths include the large sample size, randomized controlled design, intent-to-treat analyses, and the use of multiple imputation methods as these have shown improved performance over alternative approaches such as complete case analysis or single imputation methods [36]. Limitations include the lack of a 6-month follow-up for the WLC and the considerable amount of missing data at 3- and 6-month follow-up. The non-significant differences between the 3 Featback conditions should be interpreted with caution as statistical power might have been reduced due to the missing data at 3- and 6-month follow-up. The use of broad eligibility criteria can be regarded as both a strength and a limitation. The broad inclusion criteria may well have led to a study population that bears close resemblance to reality, thereby enhancing the generalizability of our findings and being consistent with the aim of an easily accessible intervention for a broad population of individuals with ED psychopathology. Alternatively, the broad inclusion criteria can be regarded as a limitation given the potential influences of variables such as the presence of comorbid disorders or the use of co-interventions on study outcome measures that were not under study control. Nevertheless, we attempted to reduce the risk of bias by acquiring detailed information on participant characteristics and external influences, so that these influences could be examined and controlled for in the analyses. Finally, the use of Web-based self-report assessments can be considered both a strength and limitation. Advantages include a reduction in research costs and being in line with the aims of the anonymous ehealth intervention: being able to remain anonymous, which lowers the barriers of seeking help and maximizing the accessibility, efficiency, and availability of health care services. Another advantage includes the minimization of the risk of bias because of the lack of face-to-face contact with participants. However, the latter might have reduced study and/or intervention commitment [9], and it resulted in the absence of a face-to-face diagnostic interview. Although we did provide a diagnostic impression of the study sample using the EDE-Q [43], it must be emphasized that the resulting classifications provide only an approximation of DSM-5 classifications as there are limitations to the use of the EDE-Q in evaluating the diagnostic criteria of ED [53].

In conclusion, an Internet-based fully automated monitoring and feedback intervention was effective in reducing psychopathology and is an interesting means of providing care for individuals with ED symptoms. Supplemental therapist support enhanced satisfaction with the intervention but did not increase its effectiveness. An interesting next step is to economically evaluate Featback with and without therapist support to determine its cost-effectiveness in comparison to a waiting list. Also, examining potential predictors, moderators, and mediators of intervention response will help to inform the field regarding for whom and how Featback work(s). A final topic for future investigation is a focus on opening the black box of therapeutic support in Internet-based interventions: what do therapists actually do when providing Web-based support and can their behavior be linked to the effectiveness of such interventions?

## Appendix

Multimedia Appendix 1 Specification of statistical models and contrast coding.

Multimedia Appendix 2 Non-imputed outcome data (means and standard deviations) in a trial investigating the effectiveness of Internet-based fully automated monitoring and feedback intervention “Featback” with different intensities of therapist support and a waiting list control condition.

Multimedia Appendix 3 Results of linear mixed model analyses comparing the effectiveness of an Internet-based fully automated monitoring and feedback intervention with and without therapist support (statistical model 2). Results are based on the pooled results of 100 multiple imputed datasets.

Multimedia Appendix 4 Results of linear mixed model analyses comparing the effectiveness of an Internet-based fully automated monitoring and feedback intervention with low-intensity (once a week) versus high-intensity (3 times a week) therapist support (statistical model 3). Results are based on the pooled results of 100 multiple imputed datasets.

## Abbreviations

ED: eating disorder
EDE-Q: Eating Disorder Examination Questionnaire
ED-QOL: ED-Related Quality of Life Questionnaire
PHQ-4: 4-item Patient Health Questionnaire
PTQ: Perseverative Thinking Questionnaire
SD: standard deviation
SEED: Short Evaluation of Eating Disorders
WLC: waiting list control

## References

[1] Berkman ND, Lohr KN, Bulik CM (2007). Outcomes of eating disorders: a systematic review of the literature. Int J Eat Disord.

[2] Hudson JI, Hiripi E, Pope HG, Kessler RC (2007). The prevalence and correlates of eating disorders in the National Comorbidity Survey Replication. Biol Psychiatry.

[3] Keel PK, Brown TA (2010). Update on course and outcome in eating disorders. Int J Eat Disord.

[4] Smink FR, van Hoeken D, Hoek HW (2013). Epidemiology, course, and outcome of eating disorders. Curr Opin Psychiatry.

[5] Kessler RC, Berglund PA, Chiu WT, Deitz AC, Hudson JI, Shahly V, Aguilar-Gaxiola S, Alonso J, Angermeyer MC, Benjet C, Bruffaerts R, de Girolamo G, de Graaf R, Maria Haro J, Kovess-Masfety V, O'Neill S, Posada-Villa J, Sasu C, Scott K, Viana MC, Xavier M (2013). The prevalence and correlates of binge eating disorder in the World Health Organization World Mental Health Surveys. Biol Psychiatry.

[6] Hart LM, Granillo M, Jorm A, Paxton Sj (2011). Unmet need for treatment in the eating disorders: a systematic review of eating disorder specific treatment seeking among community cases. Clin Psychol Rev.

[7] Becker AE, Hadley AA, Perloe A, Fay K, Striegel-Moore RH (2010). A qualitative study of perceived social barriers to care for eating disorders: perspectives from ethnically diverse health care consumers. Int J Eat Disord.

[8] Beintner I, Jacobi C, Taylor CB (2012). Effects of an Internet-based prevention programme for eating disorders in the USA and Germany--a meta-analytic review. Eur Eat Disord Rev.

[9] Aardoom JJ, Dingemans AE, Spinhoven P, Van Furth EF (2013). Treating eating disorders over the internet: a systematic review and future research directions. Int J Eat Disord.

[10] Melioli T, Bauer S, Franko DL, Moessner M, Ozer F, Chabrol H, Rodgers RF (2016). Reducing eating disorder symptoms and risk factors using the internet: A meta-analytic review. Int J Eat Disord.

[11] Aardoom JJ, Dingemans AE, Van Furth EF (2016). E-Health Interventions for Eating Disorders: Emerging Findings, Issues, and Opportunities. Curr Psychiatry Rep.

[12] Bauer S, Papezova H, Chereches R, Caselli G, McLoughlin O, Szumska I, van Furth E, Ozer F, Moessner M (2013). Advances in the prevention and early intervention of eating disorders: The potential of Internet-delivered approaches. Mental Health & Prevention.

[13] Loucas CE, Fairburn CG, Whittington C, Pennant ME, Stockton S, Kendall T (2014). E-therapy in the treatment and prevention of eating disorders: A systematic review and meta-analysis. Behav Res Ther.

[14] Andersson G, Cuijpers P (2009). Internet-based and other computerized psychological treatments for adult depression: a meta-analysis. Cogn Behav Ther.

[15] Spek V, Cuijpers P, Nyklícek I, Riper H, Keyzer J, Pop V (2007). Internet-based cognitive behaviour therapy for symptoms of depression and anxiety: a meta-analysis. Psychol Med.

[16] Baumeister H, Reichler L, Munzinger M, Lin J (2015). The impact of guidance on Internet-based mental health interventions: A systematic review. Internet Interventions.

[17] Klein B, Austin D, Pier C, Kiropoulos L, Shandley K, Mitchell J, Gilson K, Ciechomski L (2009). Internet-based treatment for panic disorder: does frequency of therapist contact make a difference?. Cogn Behav Ther.

[18] Tate DF, Jackvony EH, Wing RR (2006). A randomized trial comparing human e-mail counseling, computer-automated tailored counseling, and no counseling in an Internet weight loss program. Arch Intern Med.

[19] Kelders SM, Bohlmeijer E, Pots WT, van Gemert-Pijnen JE (2015). Comparing human and automated support for depression: Fractional factorial randomized controlled trial. Behav Res Ther.

[20] Cohen JS, Edmunds JM, Brodman DM, Benjamin CL, Kendall PC (2013). Using self-monitoring: Implementation of collaborative empiricism in cognitive-behavioral therapy. Cognitive and Behavioral Practice.

[21] Fairburn CG, Cooper Z, Shafran R (2003). Cognitive behaviour therapy for eating disorders: a “transdiagnostic” theory and treatment. Behav Res Ther.

[22] Aardoom JJ, Dingemans AE, Spinhoven P, Hakkaart-van Roijen L, Van Furth EF (2013). An Internet-based intervention for eating disorders consisting of automated computer-tailored feedback with or without supplemented frequent or infrequent support from a coach: study protocol for a randomized controlled trial. Trials.

[23] Featback.

[24] Proud2Bme.

[25] Killen JD, Taylor CB, Hayward C, Wilson DM, Haydel KF, Hammer LD, Simmonds B, Robinson TN, Litt I, Varady A (1994). Pursuit of thinness and onset of eating disorder symptoms in a community sample of adolescent girls: a three-year prospective analysis. Int J Eat Disord.

[26] Bauer S, Winn S, Schmidt U, Kordy H (2005). Construction, scoring and validation of the Short Evaluation of Eating Disorders (SEED). Eur Eat Disord Rev.

[27] Bauer S, Moessner M, Wolf M, Haug S, Kordy H (2009). ES[S]PRIT - an Internet-based programme for the prevention and early intervention of eating disorders in college students. Br J Guid Couns.

[28] Schlaken F, Wolters W, Tilanus M, van Gemert M, Hoogenhuyze C, Meijer E, Kraefft E, Brenninkmeijer M, Postel M (2010). Handbook of Online Counseling [Handboek online hulpverlening].

[29] Fairburn CG, Beglin Sj, Fairburn CG (2008). Eating Disorder Examination Questionnaire (EDE-Q 6.0). Cognitive behavior therapy and eating disorders.

[30] Berg KC, Peterson CB, Frazier P, Crow SJ (2012). Psychometric evaluation of the eating disorder examination and eating disorder examination-questionnaire: A systematic review of the literature. Int J Eat Disord.

[31] Engel SG, Wittrock DA, Crosby RD, Wonderlich SA, Mitchell JE, Kolotkin RL (2006). Development and psychometric validation of an eating disorder-specific health-related quality of life instrument. Int J Eat Disord.

[32] Kroenke K, Spitzer RL, Williams JB, Löwe Bernd (2009). An ultra-brief screening scale for anxiety and depression: the PHQ-4. Psychosomatics.

[33] Ehring T, Raes F, Weidacker K, Emmelkamp PMG (2012). Validation of the Dutch Version of the Perseverative Thinking Questionnaire (PTQ-NL). European Journal of Psychological Assessment.

[34] Hakkaart-van Roijen L, Donker M, Tiemens B (2002). Handleiding trimbos/iMTA questionnaire for costs associated with psychiatric illness (TiC-P).

[35] Doove LL, Van Buuren S, Dusseldorp E (2014). Recursive partitioning for missing data imputation in the presence of interaction effects. Computational Statistics and Data Analysis.

[36] Schafer JL, Graham JW (2002). Missing data: our view of the state of the art. Psychol Methods.

[37] Stevens J (2009). Applied multivariate statistics for the social sciences.

[38] Rubin DB (1987). Multiple Imputation for Nonresponse in Surveys.

[39] Feingold A (2015). Confidence interval estimation for standardized effect sizes in multilevel and latent growth modeling. J Consult Clin Psychol.

[40] Aardoom JJ, Dingemans AE, Slof Op't Landt MC, Van Furth EF (2012). Norms and discriminative validity of the Eating Disorder Examination Questionnaire (EDE-Q). Eat Behav.

[41] Rø Ø, Reas DL, Stedal K (2015). Eating Disorder Examination Questionnaire (EDE-Q) in Norwegian Adults: Discrimination between Female Controls and Eating Disorder Patients. Eur Eat Disord Rev.

[42] Machado PP, Martins C, Vaz AR, Conceição Eva, Bastos AP, Gonçalves Sónia (2014). Eating disorder examination questionnaire: psychometric properties and norms for the Portuguese population. Eur Eat Disord Rev.

[43] American Psychiatric Association (2013). Diagnosticstatistical manual of mental disorders (5th ed).

[44] Cuijpers P, van Straten A, Andersson G (2008). Internet-administered cognitive behavior therapy for health problems: a systematic review. J Behav Med.

[45] Kok G, Burger H, Riper H, Cuijpers P, Dekker J, van Marwijk H, Smit F, Beck A, Bockting CL (2015). The Three-Month Effect of Mobile Internet-Based Cognitive Therapy on the Course of Depressive Symptoms in Remitted Recurrently Depressed Patients: Results of a Randomized Controlled Trial. Psychother Psychosom.

[46] Berger T, Caspar F, Richardson R, Kneubühler B, Sutter D, Andersson G (2011). Internet-based treatment of social phobia: A randomized controlled trial comparing unguided with two types of guided self-help. Behav Res Ther.

[47] Rheker J, Andersson G, Weise C (2015). The role of “on demand” therapist guidance vs no support in the treatment of tinnitus via the internet: A randomized controlled trial. Internet Interventions.

[48] Mohr DC, Duffecy J, Ho J, Kwasny M, Cai X, Burns MN, Begale M (2013). A randomized controlled trial evaluating a manualized TeleCoaching protocol for improving adherence to a web-based intervention for the treatment of depression. PLoS One.

[49] Linville D, Brown T, Sturm K, McDougal T (2012). Eating disorders and social support: perspectives of recovered individuals. Eat Disord.

[50] Tregarthen JP, Lock J, Darcy AM (2015). Development of a smartphone application for eating disorder self-monitoring. Int J Eat Disord.

[51] Crow SJ, Agras WS, Halmi KA, Fairburn CG, Mitchell JE, Nyman JA (2013). A cost effectiveness analysis of stepped care treatment for bulimia nervosa. Int J Eat Disord.

[52] Mitchell JE, Agras S, Crow S, Halmi K, Fairburn CG, Bryson S, Kraemer H (2011). Stepped care and cognitive-behavioural therapy for bulimia nervosa: randomised trial. Br J Psychiatry.

[53] Berg KC, Stiles-Shields EC, Swanson SA, Peterson CB, Lebow J, Le Grange Daniel (2012). Diagnostic concordance of the interview and questionnaire versions of the eating disorder examination. Int J Eat Disord.

