# Supplementary material for: Web-Based Fully Automated Self-Help With Different Levels of Therapist Support for Individuals With Eating Disorder Symptoms: A Randomized Controlled Trial
Source: J Med Internet Res. 2016 Jun 17;18(6):e159. doi: 10.2196/jmir.5709 (PMC4930527; doi:10.2196/jmir.5709)
Supplement: Multimedia Appendix 1 [file jmir_v18i6e159_app1.pdf]

Specification of statistical models and contrast coding.

Condition contrasts (CC):

	CC1	CC2	CC3
Waiting list control	0	0	0
Featback without therapist support	1	1	0
Featback with low-intensity therapist support	1	-1	1
Featback with high-intensity therapist support	1	-1	-1

Time contrasts (TC):

	TC1	TC2	TC3
Baseline	-1	0	0
Post-intervention	1	-1	-1
3-month follow-up	0	1	0
6-month follow-up	0	0	1

Statistical model 1:

CC1 (Featback with & without therapist support versus waiting list control)

TC1 (Baseline versus post-intervention)

TC2 (Post-intervention versus 3-month follow-up)

CC1*TC1

CC1*TC2

Statistical model 2:

CC2 (Featback with therapist support versus Featback without therapist support)

TC1 (Baseline versus post-intervention)

TC2 (Post-intervention versus 3-month follow-up)

TC3 (Post-intervention versus 6-month follow-up)

CC2*TC1

CC2*TC2

CC2*TC3

Statistical model 3:

CC3 (Featback with low- intensity versus high-intensity therapist support)

TC1 (Baseline versus post-intervention)

TC2 (Post-intervention versus 3-month follow-up)

TC3 (Post-intervention versus 6-month follow-up)

CC3*TC1

CC3*TC2

CC3*TC3

Bestandsnaam: multimedia app 1
Map: P:\ehealth\ehealthdocumenten\featback\artikel\submission 6 JMIR\submission 3
Sjabloon: \\FSRC2038\SpaceProfile$\JAardoo\RESProfile\Sjablonen\Normal.dotm
Titel:
Onderwerp:
Auteur: JAardoo
Trefwoorden:
Opmerkingen:
Aanmaakdatum: 14-6-2016 10:37:00
Wijzigingsnummer: 1
Laatst opgeslagen op: 14-6-2016 10:37:00
Laatst opgeslagen door: JAardoo
Totale bewerkingstijd: 0 minuten
Laatst afgedrukt op: 14-6-2016 10:37:00
Vanaf laatste volledige afdruk
Aantal pagina's: 1
Aantal woorden: 273 (ong.)
Aantal tekens: 1.503 (ong.)
