# Supplementary material for: Web-Based Fully Automated Self-Help With Different Levels of Therapist Support for Individuals With Eating Disorder Symptoms: A Randomized Controlled Trial
Source: J Med Internet Res. 2016 Jun 17;18(6):e159. doi: 10.2196/jmir.5709 (PMC4930527; doi:10.2196/jmir.5709)
Supplement: Multimedia Appendix 2 [file jmir_v18i6e159_app2.pdf]

Non-imputed outcome data (means and standard deviations) in a trial investigating the effectiveness of Internet-based fully automated monitoring- and feedback intervention *'Featback'* with different intensities of therapist support and a waiting list control condition.

Measure	Waiting list control (*n*=90)	Featback without therapist support (*n*=87)	Featback + Low-intensity therapist support (*n*=88)	Featback + High-intensity therapist support (*n*=89)
Anorectic psychopathology (SEED-AN)				
Baseline	1.11 (0.43)	1.07 (0.37)	1.09 (0.42)	1.11 (0.41)
Post-intervention	1.13 (0.41)	1.10 (0.40)	1.14 (0.42)	1.05 (0.40)
3-Month follow-up	1.03 (0.41)	1.12 (0.42)	1.03 (0.44)	1.05 (0.36)
6-Month follow-up	n/a	1.00 (0.39)	1.01 (0.45)	1.05 (0.35)
Bulimic psychopathology (SEED-BN)				
Baseline	1.47 (0.70)	1.41 (0.66)	1.50 (0.72)	1.49 (0.63)
Post-intervention	1.45 (0.69)	1.25 (0.68)	1.27 (0.63)	1.29 (0.71)
3-Month follow-up	1.27 (0.71)	1.02 (0.67)	1.28 (0.76)	1.02 (0.70)
6-Month follow-up	n/a	0.97 (0.74)	1.25 (0.60)	0.94 (0.68)
Global ED psychopathology (EDE-Q)				
Baseline	4.05 (1.11)	4.24 (0.80)	4.43 (0.94)	3.95 (0.79)
Post-intervention	3.92 (1.14)	4.05 (1.00)	3.81 (1.18)	3.58 (1.32)
3-Month follow-up	3.85 (1.32)	3.53 (1.40)	3.57 (1.37)	3.50 (1.58)
6-Month follow-up	n/a	3.43 (1.50)	3.55 (1.56)	3.15 (1.67)
ED-related quality of life (ED-QOL)				
Baseline	2.73 (0.72)	2.75 (0.68)	2.70 (0.53)	2.65 (0.56)
Post-intervention	2.46 (0.73)	2.59 (0.69)	2.55 (0.62)	2.49 (0.69)
3-Month follow-up	2.35 (0.63)	2.14 (0.69)	2.47 (0.61)	2.22 (0.66)
6-Month follow-up	n/a	2.22 (0.79)	2.39 (0.69)	1.88 (0.73)
Symptoms anxiety & depression (PHQ-4)				
Baseline	8.14 (3.11)	8.11 (3.12)	8.48 (2.74)	8.08 (3.06)
Post-intervention	7.70 (3.39)	6.50 (3.38)	6.51 (3.53)	6.79 (3.52)
3-Month follow-up	7.41 (3.50)	5.03 (3.50)	6.07 (3.65)	6.72 (3.77)
6-Month follow-up	n/a	5.00 (3.94)	6.40 (3.65)	6.29 (3.92)
Perseverative thinking (PTQ)				
Baseline	2.78 (0.76)	2.79 (0.73)	2.86 (0.70)	2.78 (0.64)
Post-intervention	2.78 (0.86)	2.58 (0.98)	2.46 (1.04)	2.40 (1.01)
3-Month follow-up	2.52 (1.00)	2.11 (1.02)	2.29 (1.03)	2.39 (1.04)
6-Month follow-up	n/a	2.09 (1.05)	2.33 (1.07)	2.31 (1.18)

SEED=Short Examination of Eating Disorders; AN=Anorexia Nervosa; BN=Bulimia Nervosa; EDE-Q=Eating Disorder Examination Questionnaire; ED-QOL=Eating Disorder-related Quality Of Life; PHQ-4=Patient Health Questionnaire; PTQ= Perseverative Thinking Questionnaire

Bestandsnaam: multimedia app 2
Map: P:\ehealth\ehealthdocumenten\featback\artikel\submission 6 JMIR\submission 3
Sjabloon: \\FSRC2038\SpaceProfile$\JAardoo\RESProfile\Sjablonen\Normal.dotm
Titel:
Onderwerp:
Auteur: JAardoo
Trefwoorden:
Opmerkingen:
Aanmaakdatum: 14-6-2016 10:42:00
Wijzigingsnummer: 1
Laatst opgeslagen op: 14-6-2016 10:42:00
Laatst opgeslagen door: JAardoo
Totale bewerkingstijd: 0 minuten
Laatst afgedrukt op: 14-6-2016 10:42:00
Vanaf laatste volledige afdruk
Aantal pagina's: 1
Aantal woorden: 403 (ong.)
Aantal tekens: 2.219 (ong.)
