# Supplementary material for: Web-Based Fully Automated Self-Help With Different Levels of Therapist Support for Individuals With Eating Disorder Symptoms: A Randomized Controlled Trial
Source: J Med Internet Res. 2016 Jun 17;18(6):e159. doi: 10.2196/jmir.5709 (PMC4930527; doi:10.2196/jmir.5709)
Supplement: Multimedia Appendix 3 [file jmir_v18i6e159_app3.pdf]

Results of linear mixed model analyses comparing the effectiveness of an Internet-based fully automated monitoring- and feedback intervention with and without therapist support (statistical model 2). Results are based on the pooled results of 100 multiple imputed datasets.

	Time effects			Time x Condition effects		
**Measure**	*B*	*t (p)*	*95% CI*	*B*	*t (p)*	*95% CI*
Anorectic psychopathology (SEED-AN)						
Baseline to post-intervention	−0.02	−1.49 (.14)	−0.05 to 0.006	0.006	0.44 (.66)	−0.02 to 0.03
Post-intervention to 3-month follow-up	−0.007	−0.45 (.66)	−0.04 to 0.02	0.02	0.99 (.32)	−0.02 to 0.05
Post-intervention to 6-month follow-up	−0.04	−1.93 (.06)	−0.07 to .001	−0.02	−0.86 (.39)	−0.05 to 0.02
Bulimic psychopathology (SEED-BN)						
Baseline to post-intervention	−0.21	−8.07 (<.001)	−0.26 to −0.16	−0.006	−0.23 (.82)	−0.06 to 0.05
Post-intervention to 3-month follow-up	−0.08	−2.81 (.005)	−0.14 to −0.03	−0.01	−0.35 (.73)	−0.08 to 0.06
Post-intervention to 6-month follow-up	−0.19	−4.73(<.001)	−0.27 to −0.11	−0.03	−0.66 (.51)	−0.11 to 0.06
Global ED psychopathology (EDE-Q)						
Baseline to post-intervention	−0.40	−8.45 (<.001)	−0.49 to −0.31	0.05	1.02 (.31)	−0.05 to 0.15
Post-intervention to 3-month follow-up	−0.11	−2.53 (.01)	−0.20 to −0.02	−0.01	−0.23 (.82)	−0.11 to 0.09
Post-intervention to 6-month follow-up	−0.37	−5.76 (<.001)	−0.49 to −0.24	−0.01	−0.23 (.82)	−0.14 to 0.11
ED-related quality of life (ED-QOL)						
Baseline to post-intervention	−0.22	−10.51 (<.001)	−0.27 to −0.18	−0.005	−0.22 (.83)	−0.05 to 0.04
Post-intervention to 3-month follow-up	−0.09	−3.70 (<.001)	−0.14 to −0.04	−0.02	−0.71 (.48)	−0.07 to 0.03
Post-intervention to 6-month follow-up	−0.22	−7.46 (<.001)	−0.27 to −0.16	−0.01	−0.39 (.70)	−0.07 to 0.05
Symptoms anxiety & depression (PHQ-4)						
Baseline to post-intervention	−1.38	−11.76 (<.001)	−1.61 to −1.15	−0.13	−0.94 (.35)	−0.40 to 0.14
Post-intervention to 3-month follow-up	−0.50	−3.67 (<.001)	−0.76 to −0.23	−0.09	−0.58 (.56)	−0.38 to 0.21
Post-intervention to 6-month follow-up	−0.88	−5.33 (<.001)	−1.21 to −0.56	−0.15	−0.94 (.35)	−0.47 to 0.17
Perseverative thinking (PTQ)						
Baseline to post-intervention	−0.30	−8.99 (<.001)	−0.36 to −0.23	0.02	0.60 (.55)	−0.05 to 0.09
Post-intervention to 3-month follow-up	−0.13	−3.49 (.001)	−0.20 to −0.06	−0.03	−0.59 (.55)	−0.11 to 0.06
Post-intervention to 6-month follow-up	−0.23	−4.74 (<.001)	−0.33 to −0.13	−0.005	−0.09 (.93)	−0.10 to 0.09

SEED=Short Examination of Eating Disorders; AN=Anorexia Nervosa; BN=Bulimia Nervosa; EDE-Q=Eating Disorder Examination Questionnaire; ED-QOL=Eating Disorder-related Quality Of Life; PHQ-4=Patient Health Questionnaire; PTQ= Perseverative Thinking Questionnaire

Note 1: Values smaller than 0.01 are reported to 3 decimals places.

Note 2: Including the covariates age, duration of eating disorder psychopathology, and number of received psychological health care appointments.

Bestandsnaam: multimedia app 3
Map: P:\ehealth\ehealthdocumenten\featback\artikel\submission 6 JMIR\submission 3
Sjabloon: \\FSRC2038\SpaceProfile$\JAardoo\RESProfile\Sjablonen\Normal.dotm
Titel:
Onderwerp:
Auteur: JAardoo
Trefwoorden:
Opmerkingen:
Aanmaakdatum: 14-6-2016 10:43:00
Wijzigingsnummer: 1
Laatst opgeslagen op: 14-6-2016 10:46:00
Laatst opgeslagen door: JAardoo
Totale bewerkingstijd: 3 minuten
Laatst afgedrukt op: 14-6-2016 10:46:00
Vanaf laatste volledige afdruk
Aantal pagina's: 1
Aantal woorden: 483 (ong.)
Aantal tekens: 2.659 (ong.)
