# Supplementary material for: Web-Based Fully Automated Self-Help With Different Levels of Therapist Support for Individuals With Eating Disorder Symptoms: A Randomized Controlled Trial
Source: J Med Internet Res. 2016 Jun 17;18(6):e159. doi: 10.2196/jmir.5709 (PMC4930527; doi:10.2196/jmir.5709)
Supplement: Multimedia Appendix 4 [file jmir_v18i6e159_app4.pdf]

Results of linear mixed model analyses comparing the effectiveness of an Internet-based fully automated monitoring- and feedback intervention with low-intensity (once a week) versus high-intensity (three times a week) therapist support (statistical model 3). Results are based on the pooled results of 100 multiple imputed datasets.

	Time effects			Time x Condition effects		
**Measure**	***B***	***t (p)***	***95% CI***	***B***	***t (p)***	***95% CI***
Anorectic psychopathology (SEED-AN)						
Baseline to post-intervention	−0.02	−1.63 (.10)	−0.05 to 0.004	0.01	0.77 (.44)	−0.02 to 0.05
Post-intervention to 3-month follow-up	−0.01	−0.81 (.42)	−0.04 to 0.02	0.007	0.32 (.75)	−0.04 to 0.05
Post-intervention to 6-month follow-up	−0.03	−1.67 (.10)	−0.70 to .005	−0.03	−1.28 (.20)	−0.07 to 0.02
Bulimic psychopathology (SEED-BN)						
Baseline to post-intervention	−0.21	−8.48 (<.001)	−0.25 to −0.16	0.05	1.54 (.13)	−0.01 to 0.11
Post-intervention to 3-month follow-up	−0.08	−2.89 (.004)	−0.14 to −0.03	0.03	0.69 (.49)	−0.05 to 0.11
Post-intervention to 6-month follow-up	−0.18	−5.04 (<.001)	−0.25 to −0.11	0.07	1.77 (.08)	−0.007 to 0.15
Global ED psychopathology (EDE-Q)						
Baseline to post-intervention	−0.41	−9.21 (<.001)	−0.50 to −0.33	0.06	1.11 (.27)	−0.05 to 0.17
Post-intervention to 3-month follow-up	−0.11	−2.59 (.01)	−0.19 to −0.03	−0.01	−0.19 (.85)	−0.13 to 0.11
Post-intervention to 6-month follow-up	−0.36	−6.09 (<.001)	−0.48 to −0.25	0.10	1.41 (.16)	−0.04 to 0.23
ED-related quality of life (ED-QOL)						
Baseline to post-intervention	−0.22	−11.19 (<.001)	−0.26 to −0.18	0.09	3.25 (.001)	0.03 to 0.14
Post-intervention to 3-month follow-up	−0.09	−3.83 (<.001)	−0.13 to −0.04	0.04	1.44 (.15)	−0.02 to 0.10
Post-intervention to 6-month follow-up	−0.21	−8.13 (<.001)	−0.26 to −0.16	0.08	2.50 (.01)	0.02 to 0.15
Symptoms anxiety & depression (PHQ-4)						
Baseline to post-intervention	−1.34	−12.17 (<.001)	−1.56 to −1.12	−0.20	−1.33 (.18)	−0.51 to 0.10
Post-intervention to 3-month follow-up	−0.49	−3.84 (<.001)	−0.73 to −0.23	−0.23	−1.31 (.19)	−0.57 to 0.11
Post-intervention to 6-month follow-up	−0.83	−5.56 (<.001)	−1.12 to −0.54	0.09	0.52 (.61)	−0.26 to 0.44
Perseverative thinking (PTQ)						
Baseline to post-intervention	−0.30	−9.56 (<.001)	−0.36 to −0.24	0.004	0.84 (.93)	−0.08 to 0.09
Post-intervention to 3-month follow-up	−0.12	−3.56 (<.001)	−0.19 to −0.05	−0.03	−0.56 (.58)	−0.12 to 0.07
Post-intervention to 6-month follow-up	−0.23	−5.06 (<.001)	−0.32 to −0.14	0.01	0.28 (.78)	−0.09 to 0.12

SEED=Short Examination of Eating Disorders; AN=Anorexia Nervosa; BN=Bulimia Nervosa; EDE-Q=Eating Disorder Examination Questionnaire; ED-QOL=Eating Disorder-related Quality Of Life; PHQ-4=Patient Health Questionnaire; PTQ= Perseverative Thinking Questionnaire

Note 1: Values smaller than 0.01 are reported to 3 decimals places.

Note 2: Including the covariates age, duration of eating disorder psychopathology, and number of received psychological health care appointments

Bestandsnaam: multimedia app 4
Map: P:\ehealth\ehealthdocumenten\featback\artikel\submission 6 JMIR\submission 3
Sjabloon: \\FSRC2038\SpaceProfile$\JAardoo\RESProfile\Sjablonen\Normal.dotm
Titel:
Onderwerp:
Auteur: JAardoo
Trefwoorden:
Opmerkingen:
Aanmaakdatum: 14-6-2016 10:51:00
Wijzigingsnummer: 1
Laatst opgeslagen op: 14-6-2016 10:52:00
Laatst opgeslagen door: JAardoo
Totale bewerkingstijd: 1 minuut
Laatst afgedrukt op: 14-6-2016 10:52:00
Vanaf laatste volledige afdruk
Aantal pagina's: 2
Aantal woorden: 491 (ong.)
Aantal tekens: 2.703 (ong.)
